# Advances and Challenges in Viability Detection of Foodborne Pathogens

**DOI:** 10.3389/fmicb.2016.01833

**Published:** 2016-11-22

**Authors:** Dexin Zeng, Zi Chen, Yuan Jiang, Feng Xue, Baoguang Li

**Affiliations:** ^1^College of Veterinary Medicine, Nanjing Agricultural UniversityNanjing, China; ^2^Animal Quarantine Laboratory, Jiangsu Entry-Exit Inspection and Quarantine BureauNanjing, China; ^3^Shanghai Entry-Exit Inspection and Quarantine BureauShanghai, China; ^4^Division of Molecular Biology, Center for Food Safety and Applied Nutrition, United States Food and Drug Administration, LaurelMD, USA

**Keywords:** viability detection, foodborne pathogens, propidium monoazide, ethidium monoazide, PMA-qPCR, outbreaks, false positive detection

## Abstract

Foodborne outbreaks are a serious public health and food safety concern worldwide. There is a great demand for rapid, sensitive, specific, and accurate methods to detect microbial pathogens in foods. Conventional methods based on cultivation of pathogens have been the gold standard protocols; however, they take up to a week to complete. Molecular assays such as polymerase chain reaction (PCR), sequencing, microarray technologies have been widely used in detection of foodborne pathogens. Among molecular assays, PCR technology [conventional and real-time PCR (qPCR)] is most commonly used in the foodborne pathogen detection because of its high sensitivity and specificity. However, a major drawback of PCR is its inability to differentiate the DNA from dead and viable cells, and this is a critical factor for the food industry, regulatory agencies and the consumer. To remedy this shortcoming, researchers have used biological dyes such as ethidium monoazide and propidium monoazide (PMA) to pretreat samples before DNA extraction to intercalate the DNA of dead cells in food samples, and then proceed with regular DNA preparation and qPCR. By combining PMA treatment with qPCR (PMA-qPCR), scientists have applied this technology to detect viable cells of various bacterial pathogens in foods. The incorporation of PMA into PCR-based assays for viability detection of pathogens in foods has increased significantly in the last decade. On the other hand, some downsides with this approach have been noted, particularly to achieve complete suppression of signal of DNA from the dead cells present in some particular food matrix. Nowadays, there is a tendency of more and more researchers adapting this approach for viability detection; and a few commercial kits based on PMA are available in the market. As time goes on, more scientists apply this approach to a broader range of pathogen detections, this viability approach (PMA or other chemicals such as platinum compound) may eventually become a common methodology for the rapid, sensitive, and accurate detection of foodborne pathogens. In this review, we summarize the development in the field including progress and challenges and give our perspective in this area.

## Introduction

Foodborne pathogens such as *Escherichia coli* O157:H7, *Salmonella* spp., *Staphylococcus aureus, Listeria monocytogenes, Campylobacter* spp., and *Vibrio parahaemolyticus* have been a public health concern and there is a growing demand for rapid, sensitive, and accurate methods to detect these pathogens (Scallan et al., 2011). According to the Centers for Disease Control and Prevention (CDC), foodborne pathogens are responsible for more than 48 million illnesses, 128,000 hospitalizations, and 3,000 deaths in the United States each year (Scallan et al., 2011). In 2013, there was a total of 5,196 foodborne outbreaks reported in the European Union, resulting in 43,183 infected humans, 5,946 hospitalizations, and 11 deaths (Da Silva Felicio et al., 2015). The global impact of foodborne illnesses is evidenced by its significant economic impact. The costs of foodborne illness extend from the direct medical costs associated with the illness to costs incurred by the industry through product recalls, loss of consumer confidence, and litigation. It has been estimated that the aggregated annual costs of foodborne illness in the United States exceed 77 million dollars (Scharff, 2012). Given the public health and economic impact of foodborne illness, it is important to study the distribution of foodborne microbes in food production chains and develop reliable and rapid methods for pathogen detection.

Traditional culture and microscopy methods for detection of viable cells can be tedious, labor-intensive and time-consuming. Some methods enable viability to be assessed by staining techniques, such as BacLight fluorescence microscopy or acridine orange, flow cytometry coupled with dyes, and physiological tests such as for cellular respiration but do not allow for detection of specific pathogen species (Diaper and Edwards, 1994; Caron et al., 1998; Keer and Birch, 2003). These culture-based methods give rise to several challenges such as the isolation and identification of specific pathogens among a plethora of background microflora, and the detection of pathogens that occur at low levels (Sidhu and Toze, 2009). Selective media are used to reduce growth of background microorganisms, but not without introducing potential biases (Nocker et al., 2007b). Enrichment can be used to detect low level of pathogens, however, this may enable reproduction of injured cells, and subsequently overestimate pathogen density (Sidhu and Toze, 2009). On the other hand, culture-based methods encounter another issue that some human pathogens such as *Campylobacter jejuni, E. coli, Helicobacter pylori, Klebsiella pneumoniae, L. monocytogenes, Pseudomonas aeruginosa, Salmonella* Typhimurium, *Shigella dysenteriae*, and *Vibrio cholerae* may enter a “viable but non-culturable” (VBNC) physiological state, in which they are living but cannot be grown outside of their natural habitat (Lowder et al., 2000; Oliver, 2005). Furthermore, culture-based methods are also time-consuming and tedious (Nocker et al., 2007b). Molecular assays such as polymerase chain reaction (PCR) assays are rapid, sensitive, however, they may overestimate viable cell numbers due to amplification of DNA from dead cells and extracellular DNA within samples (Rudi et al., 2005), and thus may lead to unnecessary product recalls and economic losses (Liu and Mustapha, 2014). Therefore, accurate detection of viable bacteria in foods is critical and necessary in assessing the risk for foodborne outbreaks because only live pathogens constitute the risk of foodborne outbreaks.

Currently, two techniques are available for viability detection of foodborne pathogens. The first one is based on the detection of mRNA by using reverse-transcriptase PCR (RT-PCR). It is based on that bacterial transcripts are sensitive to degradation by intra- and extracellular RNases; and mRNA levels should rapidly decline after cell death. Hence, mRNA would only be limited to the viable cells within the population. The development of RT-PCR assays to detect foodborne pathogens such as *E. coli* O157:H7 (Ju et al., 2016) utilized this premise. However, this approach is affected negatively by a few factors. First, it requires expression of the target gene(s), which may vary under stress conditions. Second, handling RNA is tedious and cumbersome due to its liability to contamination. Overall, the use of RT-PCR assay is more adapted for gene expression studies than as a detection means for foodborne pathogens (Barbau-Piednoir et al., 2014). Third, some reports have concluded that mRNA disappears quickly after cell death, while others suggest that transcripts can persist for extended lengths of time (Ju et al., 2016).

The other technique for viable cell detection is an approach that uses PCR method in conjunction with biological dyes, ethidium monoazide (EMA) and propidium monoazide (PMA, a derivative of ethidium bromide). The approach can specifically detect DNA from cells with intact cell/wall membranes and the viability discrimination is based on the characteristics of EMA and PMA. These biological dyes are positively charged molecules and thus are excluded by intact, negatively charged, bacterial cell-walls, but can enter bacteria with compromised cell-wall/membranes (Nocker and Camper, 2006).

**FIGURE 1:**
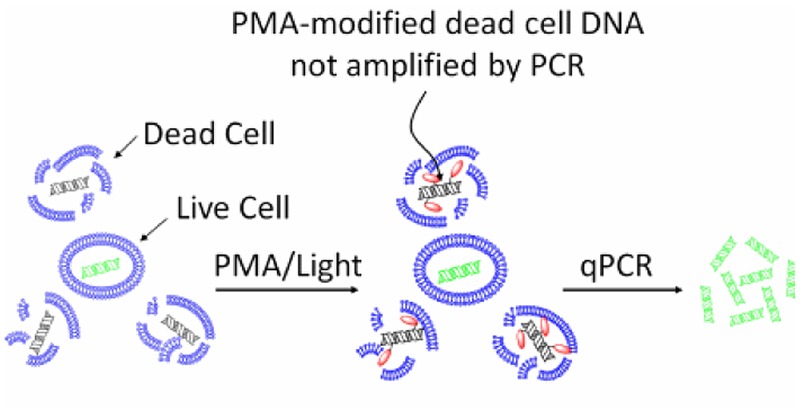
**Principle of selective detection of viable cells using PMA dye (https://biotium.com/product/pmatm-dye-20mm-in-h2o/)**.

The mechanism of action of EMA/PMA has not been fully elucidated yet, but it could be as the result of a combination of following factors, (i) when a EMA or PMA solution is added to a mixture of intact and membrane-compromised cells, the chemical can selectively enter only the compromised cells; (ii) once inside the cell, the dye intercalates into nucleic acids and the presence of an azide group allows for a cross-linking between the dye and the DNA after exposure to strong visible light; (iii) the light leads to the formation of a highly reactive nitrene radical, which can react with any organic molecule in its proximity including the bound DNA; (iv) this modification strongly inhibits the sequential DNA amplification in PCR; and (v) at the same time, when the cross-linking occurs, the light reacts with unbound excess dye with water molecules and the resulting hydroxylamine is no longer reactive, so the DNA from cells with intact membranes is not modified in the DNA extraction (Nocker et al., 2009). Therefore, by this mechanism, EMA or PMA can preferably intercalate DNA of the dead cells and thus prevent subsequent DNA amplification of dead cells by PCR as illustrated by **Figure 1**.

EMA/PMA-PCR assays have been applied to the detection of a variety of microbes, including bacterial cells and spores, fungi, viruses, and yeast in food and the environment (Agusti et al., 2010; Andorra et al., 2010; Josefsen et al., 2010; Fittipaldi et al., 2011; Li and Chen, 2012, 2013; Alonso et al., 2014; Law et al., 2015b). Recently, EMA/PMA-PCR assays have also been applied in bacterial studies on clinical samples, suggesting that this approach may have a potential alternative to diagnosis by microscopy and culture, and in clinical settings (Rogers et al., 2008; Miotto et al., 2012) or in new drug development (Caldas et al., 2014; Oliveira-Silva et al., 2015). In the article, we mainly focused on the application of this biological dye EMA/PMA in differentiation of viable cells of foodborne pathogens, *E. coli* O157:H7, *Salmonella, S. aureus, L. monocytogenes, Campylobacter*, and *V. parahaemolyticus* in foodborne pathogens and summarize the developments in this area.

Although EMA/PMA behaves nearly identically as intercalating stains, the two dyes differ in regard to their permeation through cell membranes. EMA, due to its chemical composition, is slightly more efficient in signal suppression than PMA, however, PMA is more effective than EMA in terms of live and dead discrimination. Numerous studies have used the EMA/PMA approach to detect viable cells of foodborne pathogens, and such work has yielded valuable data on the EMA/PMA efficiency and influence factors. The factors associated with efficiency of the viability detection includes the type and concentration of dye, concentration of organisms, type of food matrices, ratio between viable and dead cells, length of the PCR amplicon, physical condition of the sample, and light exposure conditions. In the study, we reviewed the development in application of the biological dyes, PMA/EMA, in differentiation of viable cells of foodborne pathogens including *E. coli* O157:H7, *Salmonella, S. aureus, L. monocytogenes, Campylobacter*, and *V. parahaemolyticus*; and we also discussed the challenges in using this approach and proposed strategies to remedy the drawbacks of this approach.

## Application of EMA/PMA in Differentiation of Viable Cells of *E. coli* O157:H7 in Food

**FIGURE 2:**
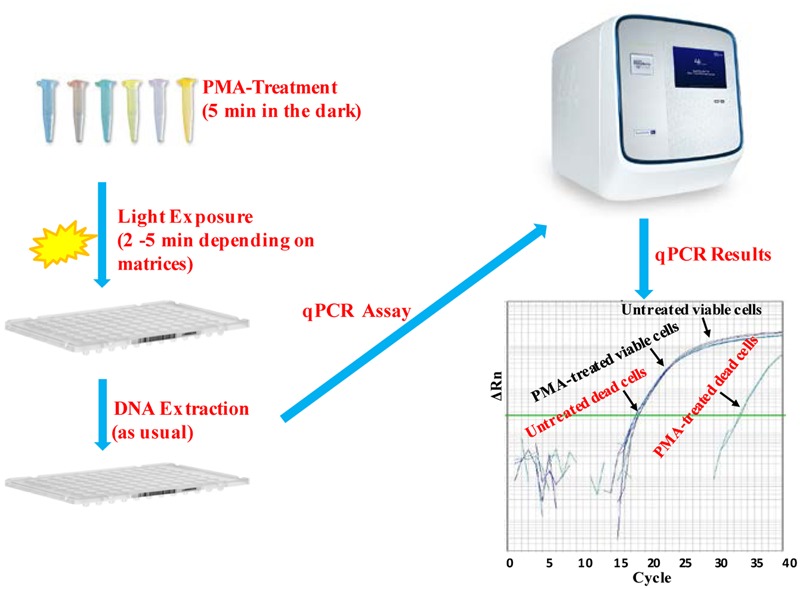
**Scheme of PMA treatment and PMA-qPCR assay**.

*Escherichia coli* O157:H7 is one of the most notorious foodborne pathogens, with an infectious dose of as low as a few hundred cells (Karmali, 2004). Beef, dairy products, juices, and fresh produce are foods that are often associated with *E. coli* O157:H7 outbreaks (Liu and Mustapha, 2014). Recently, numerous studies have applied EMA/PMA for differentiation of viable cells of *E. coli* O157:H7 in foods. Li and Chen (2012) selected ORF Z3276 as a unique detection target and applied PMA treatment to qPCR to accurately detect viable cells of *E. coli* O157:H7 in beef. They compared the different concentrations of PMA on the signal suppression of dead cells and found 50 μM PMA was the best concentration, which yielded strong signal suppression of dead cells and did not affect signal of viable cells (**Table 1**). In addition, the light exposure time was optimized at 2 min (**Table 2**). Subsequently, they optimized the PMA treatment conditions as the following: live/dead cell mixtures were added to PMA to a final concentration of 50 μM and incubated at room temperature in the dark for 5 min; the PMA-treated samples were exposed to a 650-W halogen light source, 20 cm from the samples for 2 min for the photo-induced cross-linking. After that the samples were subjected to regular DNA purification procedures and qPCR as illustrated in **Figure 2**. They demonstrated that this PMA-qPCR assay could detect 8 × 10^1^ CFU/g mixed with 8 × 10^7^ dead cells/g *E. coli* O157:H7 cells in spiked beef samples with an 8-h enrichment and that PMA treatment did not significantly affect the amplification of DNA from viable cells. In comparison, an EMA-qPCR assay could only detect at the 10^3^ CFU/g *E. coli* O157:H7 cells in spiked beef samples after 8-h enrichment but failed to detect at 10^1^ or 10^2^ CFU/g *E. coli* O157:H7 cells in spiked beef samples with an 8-h enrichment (Wang et al., 2009). In comparison of the sensitivity of the two assays, Li and Chen (2012) attribute their enhanced sensitivity to three factors: first, the higher sensitivity of the PMA-qPCR can be attributed to the higher sensitivity of this qPCR assay itself; second, it may be due to the improved PMA treatment, as indicated by the smaller differences in *C_T_* values (0.5 *C_T_* value) between the PMA-treated and untreated viable cells; and third, PMA is more selective than EMA in inhibiting DNA amplification from dead cells. It is worth noting that the PMA-qPCR assay developed by Li and Chen (2012) has been well accepted by the scientific community and industry. Recently, a commercial kit has been developed based on Li and Chen’s findings by a U.S. company for detection of viable cells of *E. coli* O157:H7^1^.

**Table 1:** Effect of different concentrations of PMA on signal suppression of dead cells of *E. coli* O157:H7 in PMA-qPCR^a^.

Cells	Concentration of PMA (μM)			
	0	25	50	100
Viable	19.84 ± 0.13^b^	19.47 ± 0.09	19.03 ± 0.31	19.07 ± 0.16
Dead	19.02 ± 0.12	32.63 ± 0.07	34.04 ± 0.25	34.96 ± 0.22

*^a^Two minutes was used for light exposure.*

*^b^The average of *C_T_* values o f triplicate.*

**Table 2:** Light exposure influence on PMA treatment of signal suppression of dead cells of *E. coli* O157:H7 in PMA-qPCR^a^.

Light exposure (min)	*C_T_* value ± SD^b^	
	Dead cells	Viable cells
0.0	29.79 ± 0.31	24.73 ± 0.35
0.5	30.32 ± 0.21	24.44 ± 0.21
1.0	29.78 ± 0.25	24.95 ± 0.59
2.0	30.21 ± 0.75	25.20 ± 0.48
4.0	30.21 ± 0.38	25.54 ± 0.47
No PMA control	24.32 ± 0.57	23.24 ± 0.12

*^a^PMA concentration used in the experiment was 50 μM.*

*^b^The average of *C_T_* values of triplicate.*

Besides qPCR, researchers have also applied PMA/EMA to loop-mediated isothermal amplification (LAMP; Chen et al., 2011; Zhao et al., 2013). Chen et al. (2011) developed PMA-LAMP to detect *Salmonella* in produce and Zhao et al. (2013) combined PMA with LAMP to evaluate the inactivation effect on *E. coli* O157:H7 by slightly acidic electrolyzed water. Zhao et al. (2013) achieved detection limit of 1.6 × 10^2^ CFU of *E. coli* O157:H7 per reaction by using *rfbE* gene as target gene and 3 μg/ml PMA as final concentration to treat the samples. Compared with PMA-qPCR, this PMA-LAMP assay had lower sensitivity, but it was more economical to run; it is particularly suitable for resource-limited labs to conduct large-scale detection.

Liu and Mustapha (2014) developed a PMA-qPCR assay for detection viable cells of *E. coli* O157:H7. They used 25 μM PMA to treat the cell mixtures with 10-min intensive light exposure. In the qPCR, the *uidA* gene and TaqMan were used. This PMA-qPCR assay could detect as low as 10^2^ CFU/ml viable *E. coli* O157:H7 in pure culture and 10^5^ CFU/g in ground beef in the presence of 10^6^/g of dead cells. With an 8-h enrichment, 1 CFU/g viable *E. coli* O157:H7 in ground beef was detectable without interference from 10^6^ dead cells/g (Liu and Mustapha, 2014). Additionally, other groups have also used PMA/EMA-qPCR to detect viable cells (Luo et al., 2010; Dinu and Bach, 2011; Elizaquivel et al., 2012; Liu et al., 2014). In contrast, in comparison of four different methods, culture, qPCR, RT-PCR, PMA-qPCR for quantitative detection of viable cells of *E. coli* O157:H7 in plant matrices, Ju et al. (2016) found that neither RT-PCR (with 2 log reduction) nor PMA-qPCR (with 3 log reduction) could efficiently suppress the DNA signals from dead cells.

## EMA/PMA in Differentiation of Viable Cells of *Salmonella* in Food

*Salmonella* infections represent a considerable global burden with significant health and economic impacts. Salmonellosis is most often attributed to the consumption of contaminated foods such as poultry, beef, pork, eggs, milk, seafood, nut products, and fresh produce (Scallan et al., 2011). There is a need for the development of more sensitive, rapid, and inexpensive methods for detection of this pathogen in foods (Techathuvanan and D’Souza, 2012; Kokkinos et al., 2014). Recently, several studies applied EMA/PMA to PCR to detect viable cells of foodborne pathogens including *Salmonella* spp. in foods. However, it was noticed in some studies that sometimes the suppression of DNA signal of dead cells was incomplete. To address that issue, an efficient PMA-qPCR assay was developed by targeting a conserved region of the *invA* gene of *Salmonella* in conjunction with PMA treatment for detection of DNA from viable cells of *Salmonella* in food (Li and Chen, 2013). In that study, Li and Chen (2013) systematically compared efficiency of five different-sized amplicons ranging from 65 to 260 bp in signal suppression of dead cells in qPCR. The most efficient amplification was detected with Amplicon A (65 bp) with a *C_T_* value of 17.75, whereas the least amplification efficiency was found with Amplicon E (260 bp) with a *C_T_* value of 21.19. These data clearly demonstrated that the Amplicon D (130-bp) was the optimized amplicon in the PMA-qPCR assay. With the Amplicon D, the authors not only attained superior qPCR amplification efficiency with a *C_T_* value of 18.34, but also achieved high signal suppression of dead cells in qPCR with a *C_T_* value of 13.18 as shown in **Table 3**. Furthermore, this PMA-qPCR assay was capable of detecting live *Salmonella* cells in live/dead cell mixtures. In addition, the level of sensitivity achieved was 30 CFU/g live *Salmonella* cells from enriched spiked spinach samples as early as 4 h.

**Table 3:** Effect of amplicons of different length on signal suppression of *Salmonella* dead cells by PMA-qPCR.

Amplicon	Sequence of primers or probe (5′–3′)	Position in *invA*^a^	Amplicon length (bp)	*C_T_* value		Signal suppression (Δ*C_T_*)^b^ (PMA efficiency)
				PMA-treated (PMA effect)	Untreated (qPCR efficiency)	
A	Forward 5′-CGTTTCCTGCGGTACTGTTAATT	197–219	65	23.81	17.75	6.06
	Reverse 5′-ACGACTGGTACTGATGATCGATAATGC	261–238				
	Probe FAM-CCACGCTCTTTCGMGBNFQ	221–233				
B	Forward 5′-CGTTTCCTGCGGTACTGTTAATT	197–219	97	29.96	18.41	11.55
	Reverse 5′-ATTTCACGGCATCGGCTTCAATC	293–270				
	Probe FAM-CCACGCTCTTTCGMGBNFQ	221–233				
C	Forward 5′-CGTTTCCTGCGGTACTGTTAATT	197–219	119	33.38	20.54	12.84
	Reverse 5′-GAATTGCCCGAACGTGGCGATAAAT	315–292				
	Probe FAM-CCACGCTCTTTCGMGBNFQ^d^	221–233				
**D^c^**	**Forward 5′-CGTTTCCTGCGGTACTGTTAATT**	**197–219**	**130**	**31.52**	**18.34**	**13.18**
	**Reverse 5′-TCGCCAATAACGAATTGCCCGAAC**	**326–303**				
	**Probe FAM-CCACGCTCTTTCGMGBNFQ**	**221–233**				
E	Forward 5′-CGTTTCCTGCGGTACTGTTAATT	197–219	260	35.53	21.19	15.44
	Reverse 5′- GACCACGGTGACAATAGAGAAG	456–435				
	Probe FAM-CCACGCTCTTTCGMGBNFQ	221–233				

*^a^*invA* gene sequence is from GenBank accession number M90846.*

*^b^Δ*C_T_* value was created by (*C_T_* value of PMA-treated dead cells -*C_T_* value of untreated dead cells).*

*^c^Bold-faced information is for the amplicon selected as the optimized amplicon in PMA-qPCR.*

It is worth noting that the optimal sized amplicon (130 bp) in the qPCR is quite different to that previously reported (Luo et al., 2010). In that work to detect viable cells of *E. coli* O157:H7, they found that PMA cannot efficiently exclude the DNA of the dead cells when the sizes of amplicons smaller than 190 bp were targeted by PCR. This is a significant size difference in designing amplicons for qPCR because the amplification efficiency of qPCR is affected by the sizes of amplicons and most of the qPCR instruments work best with amplicons under 150-bp. Therefore, selecting the right-sized amplicons for qPCR is a key factor to successfully detect viable foodborne pathogens (Li and Chen, 2013). Additionally, in the last few years, several more studies addressed the technical issues with the PMA-qPCR for detection of viable cells of *Salmonella* in food (Nocker et al., 2009; Liang et al., 2011; Banihashemi et al., 2012; Elizaquivel et al., 2012; Yang Y. et al., 2013; Barbau-Piednoir et al., 2014; Wu et al., 2015).

In order to improve the efficiency of PMA treatment, scientists have tried adding sarkosyl (0.2%) to the PMA solution and found that 0.2% sarkosyl increased PMA’s penetration to the dead cells with little effect on the viable cells (Wang et al., 2014; Li et al., 2015). Recently, a multiplex PMA-qPCR was developed to viable *Legionella pneumophila, S.* Typhimurium, and *S. aureus* in environmental waters (Li et al., 2015). The authors could detect *S.* Typhimurium and *S. aureus* with 3 CFU per reaction in their multiplex PMA-qPCR assay and further applied the assay to detect the multiple pathogens from rivers, canals, and tap water samples after simple water pretreatment. In the study, these authors optimized PMA treatment conditions as following: 30 μM PMA with 20 min of dark incubation and 10 min of light exposure. Scientists have also applied PMA approach to LAMP to detect viable cells of *Salmonella* in food. Chen et al. (2013) found PMA-LAMP was able detect viable cells in food with the detection limits comparable to that of PMA PMA-qPCR.

Besides PMA, EMA has been applied in viable cell detection of *Salmonella* in food. Wu et al. (2015) combined the EMA with LAMP to detect viable cells of *Salmonella*. They found that the concentration EMA is critical in effecting the DNA amplification of viable cells, i.e., if the EMA concentration used was <8.0 μg/ml, the DNA amplification of viable cell was not affected, otherwise, it would be significantly affected (Wu et al., 2015).

## EMA/PMA in Differentiation of Viable Cells of *Campylobacter* in Food

Campylobacteriosis remains one of the most commonly reported bacterial foodborne disease in humans worldwide (Aase et al., 2000; Banihashemi et al., 2012). The incidence of campylobacteriosis has risen, with more than 200,000 confirmed cases in the European Union reported each year (Anonymous, 1996; D’Agostino et al., 2004). *Campylobacter* infections are clinically manifested by diarrhea, fever, and abdominal cramps, and, in certain cases, may be followed by long-term sequelae such as Guillain–Barré syndrome or reactive arthritis (Anonymous, 1996). Rapid pathogen detection is imperative for food manufacturers, public health agencies and clinicians alike. The sensitivity and specificity of detection of *Campylobacter* by PCR have been validated in the field. However, only viable *Campylobacter* cells can cause diseases and PCR or qPCR are not able to differentiate dead and viable cells.

To address this shortcoming of PCR, EMA/PMA has been combined with qPCR to detect viable cells of *Campylobacter*. Josefsen et al. (2010) developed a PMA-qPCR assay to detect the three major foodborne *Campylobacter* species (*C. jejuni, Campylobacter coli*, and *Campylobacter lari*) and found the PMA-qPCR quantification compared favorably with direct culture-based detection of *Campylobacter* in their study. The limit of detection of PMA-qPCR reached 10^2^ CFU/g in the presence of dead cells. The specificity of the PMA-qPCR method was 100%, and it was shown to be more sensitive compared to the culture-based method. Eight chicken samples in that study were found to be *Campylobacter* positive by PMA-qPCR but not by culture (Josefsen et al., 2010). These PMA-QPCR results can be regarded as true positives due to the target-specific DNA probe-based PCR response according to ISO 20838 (Anonymous, 1996).

Traditional culture-based detection of *Campylobacter* bacteria, including enrichment, isolation, and confirmation, is a time-consuming procedure. Furthermore, bacterial cells may enter a VBNC state in which they may have the potential to cause human infection but are not detected by the culture method (Rollins and Colwell, 1986). Josefsen et al. (2010) developed a PMA-qPCR method to detect the infectious potential of the VBNC state. They found that the PMA-qPCR method was effective in assessing the risk of *Campylobacter* contamination including the infectious potential of the VBNC state cells in chicken carcass rinse. Banihashemi et al. (2012) also used PMA treatment and conventional PCR to tackle the issue of VBNC *Campylobacter* cells in food samples. Long amplicons were used in the PMA-PCR. The authors found when the length amplicon was <200 bp, the signal from DNA of the dead cells was not completely excluded, whereas, the length amplicon was >1.5k bp, the signal from DNA of the dead cells was completely excluded in the PMA-PCR assay (Banihashemi et al., 2012). Furthermore, the authors found that the signal of the DNA of the dead cells caused by UV-irradiation cannot be not excluded by PMA treatment (Banihashemi et al., 2012).

However, Seinige et al. (2014) gave a quite different view to the one that Flekna et al. (2007a) gave. Seinige et al. (2014) believed that EMA-qPCR was a suitable method for detection of viable *Campylobacter* from water samples, but the isolation technique and the type/quality of the water sample may impact the results. In general, most researchers preferred using PMA-qPCR than EMA-qPCR to detect viable cells *Campylobacter*.

## EMA/PMA in Differentiation of Viable Cells of *Vibrio parahaemolyticus* in Food

*Vibrio parahaemolyticus*, a major foodborne pathogen known to cause gastroenteric infections, is often isolated from seawater, sediment, and a variety of seafood including oyster, clam, scallop, octopus, shrimp, crab, lobster, crawfish (Shen et al., 2009; Letchumanan et al., 2014). *V. parahaemolyticus* can cause diarrhea, vomiting, abdominal cramps, and, in rare cases, fever (Lin et al., 2005). Conventional culture-based techniques are laborious and time consuming. qPCR is rapid and sensitive, but its inability to discriminate between live and dead cells limits its applications. Therefore, EMA/PMA was used to combine qPCR assay to detect viable cells of *V. parahaemolyticus.* But so far only limited reports have been available reporting detection of viable cells of *V. parahaemolyticus* using EMA/PMA approach (Zhu et al., 2012; Fakruddin et al., 2013; Zhang et al., 2015a). Zhu et al. (2012) used PMA-qPCR to detect viable cells of *V. parahaemolyticus* from seafood. In comparison to culture-based methods, PMA-qPCR demonstrated advantage in detection of viable cells of *V. parahaemolyticus*. The authors used *V. parahaemolyticus* strains of different serotypes and 120 seafood samples to evaluate the sensitivity and specificity of the PMA-qPCR assay. They found that the sensitivity of the PMA-qPCR was 12 *V. parahaemolyticus* CFU per reaction for seafood samples, and the amount of DNA of pure culture samples was equivalent to 1.2 CFU per reaction. In addition, they found that 8 μM of PMA was the optimal concentration for PMA treatment of *V. parahaemolyticus* samples and PMA treatment became incomplete if the turbidity of the bacterial culture was over 10 Nephelometric Turbidity Unit (NTU) or OD_600__nm_ greater than 0.8. Although the authors believed the PMA-qPCR was an effective tool for producing reliable quantitative data on viable *V. parahaemolyticus* in raw seafood (Zhu et al., 2012), there is more work needed to be done before the PMA-qPCR method can be widely used in detection of viable cells of *V. parahaemolyticus* in food.

## EMA/PMA in Differentiation of Viable Cells of *Staphylococcus aureus* in Food

*Staphylococcus aureus*, a spherical and Gram-positive bacterium, is a major cause of skin, soft tissue, respiratory, bone, joint, and endovascular disorders (Lowy, 1998). It has also been recognized as a pathogen that causes outbreaks of food poisoning (Zhang et al., 2015b). *S. aureus* can contaminate a variety of foods such as salad, cheese, milk, fish, and meat (Alarcon et al., 2006; Vazquez-Sanchez et al., 2014). Routine detection of *S. aureus* in food is usually carried out by traditional methods based on the use of selective media (e.g., Baird–Parker agar) for direct enumeration or the recovery of isolates after enrichment in selective broth for 24–48 h at 37°C. Subsequently, the suspected colonies that are positive for DNase, and then coagulase production should be tested. This conventional method takes from 5 to 6 days and has low sensitivity and specificity (Alarcon et al., 2006). Hence, conventional method may underestimate the level of contamination (Zhang et al., 2015b).

In the last two decades, numerous PCR-based methods have been developed for the detection of foodborne pathogens to replace the time-consuming culture-based classical techniques (Johnson et al., 1991; Wilson et al., 1991; Hein et al., 2001, 2005; Verhoeven et al., 2012). The inability of PCR to differentiate between viable and dead cells is one of its major limitations (Nocker and Camper, 2006; Wang and Levin, 2006; Liu et al., 2012). To remedy this drawback of PCR, Kobayashi et al. (2009) combined the PMA treatment with qPCR to detect viable cells of *S. aureus*. They found that the PMA-qPCR assay inhibited the amplification of DNA from dead bacterial cells, and the qPCR results reflected the number of viable bacteria without being impacted by the presence of the dead bacteria. This approach of combining qPCR with PMA treatment has promise to limit false-positive PCR results when used to diagnose infections, but needs to be further validated in clinical samples (Kobayashi et al., 2009). Later on, Martinon et al. (2012a,b) also applied the PMA-qPCR approach to detect viable cells of *S. aureus* and other pathogens to assess the hygienic status of food contact surfaces within a commercial frozen meal factory. By comparison of plate counts, qPCR, PMA-qPCR, and Reagent D-qPCR, they found that the results from PMA-qPCR were slightly higher than those derived from plate counts. The authors believed that the PMA-qPCR results may reflect the real bacterial number in light of the presence of VBNC among bacterial populations (Martinon et al., 2012a). Zhang et al. (2015b) combined PMA with qPCR for selective detection of viable *S. aureus* in milk power and meat products and found that the PMA-qPCR assay was more specific and sensitive than conventional PCR, and the limit of detection was 3.0 × 10^2^ CFU/g in spiked milk powder. The data indicated that the PMA treatment effectively eliminated the DNA amplification signals from dead cells but had little effect on viable cells (Zhang et al., 2015b).

## EMA/PMA in Differentiation of Viable Cells of *Listeria monocytogenes* in Food

*Listeria monocytogenes* is one the most habitually investigated foodborne pathogens, whereas the *L. monocytogenes* outbreaks had the highest proportion of hospitalized cases as well as the highest proportion of deaths registered in the European Union (Anonymous, 2011; Law et al., 2015a). Flekna et al. (2007b) combined EMA with qPCR to detect viable cells of *C. jejuni* and *L. monocytogenes*. The authors tried to use different concentrations (1–100 μg/ml) EMA to treat viable and dead cells and concluded that EMA influences not only dead but also viable cells of *C. jejuni* and *L. monocytogenes*. Thus, EMA/real-time PCR is a poor indicator of cell viability (Flekna et al., 2007b). Scientists have compared EMA-qPCR with PMA-qPCR in detection of viable cells of *L. monocytogenes* (Pan and Breidt, 2007). In order to thoroughly evaluate the two dyes, the authors compared the influence of the environmental factors such as temperature. They found that the effect of EMA on viable cells correlated with the temperature used to treat the cells, whereas PMA did not show any effect on viable cells in regard to the temperature changes in the treatment. Furthermore, the authors found that the PMA-qPCR could be used for quantification of viable cells of *L. monocytogenes* in suspensions in which the ratio of dead cells to viable cells was no more than 10^4^ and the concentration of live cells was no less than 10^3^ CFU/ml. Compared with EMA, PMA was not found to penetrate live cells, as determined by the toxicity of the two dyes (Pan and Breidt, 2007). However, when other researchers applied the PMA-qPCR in detection of viable cells from food matrix, they had slightly different opinions on this assay. For example, Elizaquivel et al. (2012) developed a multiplex PMA-qPCR to detect viable cells of *E. coli* O157:H7, *L. monocytogenes*, and *Salmonella* from fresh-cut vegetables. The authors noted that when salad with high concentration of *L. monocytogenes*, PMA treatment cannot completely exclude the influence of the dead cells. Nevertheless, their data demonstrate that PMA-qPCR is a suitable technique for the detection and quantification of viable pathogens in fresh-cut vegetables at the levels normally found in vegetable samples (Elizaquivel et al., 2012).

Yang J. et al. (2013) have combined PMA with conventional PCR to detect viable cells of *L. monocytogenes*. The authors developed a multiplex PCR to simultaneously detect viable cells of *S.* Typhimurium, *E. coli* O157:H7, and *L. monocytogenes* in food products. In order to improve the sensitivity of the assay, magnetic nanobeads-based immunomagnetic separation was used to concentrate the target bacterial cells. Consequently, their results showed the detection limit of 8.4 × 10^3^ CFU/g for *L. monocytogenes* in spiked food products (lettuce, tomato, and ground beef) (Yang J. et al., 2013). In a study comparing EMA with PMA in live-dead cell samples of four Gram-negative and four Gram-positive bacterial species, Nocker and Camper (2006) found PMA more impermeable to viable cells.

## Which Dye Works Better for Viability Detection of Foodborne Pathogens?

Cawthorn and Witthuhn (2008) documented PMA more membrane-impermeant compared with EMA in a study for selective detection of viable *Enterobacter sakazakii* cells. It was concluded that EMA was less effective than PMA in selective amplification of DNA from viable cells and PMA was a useful alternative (van Frankenhuyzen et al., 2011). Besides viable cells of bacterial pathogens (Nocker and Camper, 2006, 2009; Nocker et al., 2007a; Pan and Breidt, 2007; Luo et al., 2010; Li and Chen, 2012, 2013), the PMA-qPCR has been applied to detect viable cells of fungi (Vesper et al., 2008), parasites (Brescia et al., 2009; Cancino-Faure et al., 2016), and viruses (Fittipaldi et al., 2010; Parshionikar et al., 2010; Kim and Ko, 2012; Sanchez et al., 2012; Coudray-Meunier et al., 2013; Fuster et al., 2016).

## Limitations of the PMA Approach and the Remedies to Address the Issues

Like any other technologies or assays, PMA-qPCR has its limitations in detection of viable cells of foodborne pathogens in foods. PMA-PCR was first reported to effectively exclude the signal of dead bacteria (Nocker et al., 2006; Josefsen et al., 2010). Later, this approach was adapted by many scientists to detect viable cells of various foodborne pathogens (Cawthorn and Witthuhn, 2008; Josefsen et al., 2010; Liang et al., 2011; van Frankenhuyzen et al., 2011; Banihashemi et al., 2012; Li and Chen, 2012, 2013; Mamlouk et al., 2012; Soejima et al., 2012; Dinu and Bach, 2013; Singh et al., 2013; Yang Y. et al., 2013; Ditommaso et al., 2015; Li et al., 2015; Santiago et al., 2015; Cattani et al., 2016). The majority of the studies demonstrated that PMA-PCR effectively suppressed the signal of DNA from the dead cells. On the other hand, it was also found that PMA treatment does not always lead to complete removal of the qPCR signal of dead bacteria. Fittipaldi et al. (2010) summarized that incomplete suppression of the signal from dead will occur if (i) the amplicon size of the qPCR assay is short (Luo et al., 2010; Li and Chen, 2012; Schnetzinger et al., 2013); (ii) the target bacteria is at high concentration (Elizaquivel et al., 2012; Zhu et al., 2012; Li and Chen, 2013; Pacholewicz et al., 2013); (iii) the concentration of Mg_2_^+^ in the PCR reaction is not adapted (Nocker et al., 2006); or (iv) the fat content of food sample is high (Yang et al., 2011), and may also vary according to the “killing” treatment (Nocker et al., 2007a; Kobayashi et al., 2010; Yang et al., 2011; Liang and Keeley, 2012). Additionally, the turbidity of food samples may hamper light penetration and samples dilution is required for a thorough light exposure. Such dilution practically restricts the capacity of sample preparation and consequentially, it has to resort to extrapolation method to analyze the results, making it less accurate (van Frankenhuyzen et al., 2011).

With the downsides in using the PMA approach being recognized by scientists, the remedies have been proposed to address these issues. For example, “activity-labile compounds” as a possible alternative for PMA treatment was suggested (Nocker and Camper, 2009). Soejima et al. (2016) recommended to use platinum compounds. Platinum metals can be chelated by nucleic acid ligands in mammalian cells (Rosenberg et al., 1965, 1969; Lovejoy et al., 2008; Serrano et al., 2011). Platinum compounds do not depend on visible light to function and they are inexpensive. Using Pt compounds in viable detection can avoid the laborious procedures in PMA treatment (Cimino et al., 1991; Rudi et al., 2005; Nocker et al., 2006). More recently, Soejima et al. (2016) compared five platinum compounds with PMA in viable cell detection and indicated that this platinum-PCR method completely suppressed the signal of dead cells and enabled the specific detection of viable coliforms in milk at a concentration of 5–10 CFU/ml specified by EU/USA regulations after a 4-h process.

## Conclusion and Perspective

Obviously, culture-based methods cannot match the challenges that the foodborne pathogens pose to the food safety and public health. There is a great demand for rapid, sensitive, specific, and accurate methodologies for pathogen detection in foods. Molecular assays such as PCR and LAMP methods have been demonstrated huge advantages in sensitivity, specificity, and speed. However, a major drawback of these assays is that their inability to differentiate viable and dead cells may overestimate risk of contamination of foodborne pathogens. To circumvent this shortcoming, recently, scientists have combined EMA/PMA to PCR or LAMP for accurate detection of viable cells of foodborne pathogens in foods. Numerous studies have used this approach for detection of viable cells of various foodborne pathogens, including *E. coli* O157:H7, *Salmonella, S. aureus, V. parahaemolyticus, L. monocytogenes*, and *Campylobacter*, and achieved various degree of success in surpassing the signals of DNA of the dead cells in the detection assay. In general, this PMA approach is the most practical means for detection of viable cells of foodborne pathogens.

As pathogen detection method, PCR, qPCR, or LAMP method each shows its advantages and shortcomings. PMA-qPCR is the most commonly used technique in foodborne detection because it is rapid, specific, sensitive, and quantitative. Although the sensitivity of PMA-LAMP is slightly lower than that of PMA-qPCR, it is simpler, more economic, and particularly suitable for the need of pathogen detection of resource-limited institutions.

PMA is more preferably used dye in viable cells detection of foodborne pathogens compared with EMA, which has been shown not only penetrates compromised membranes of dead cells but also penetrate membranes of viable cells, leading false negative results by numerous studies. While PMA-qPCR assays have been successfully applied to various foodborne pathogens, the drawbacks of this approach have been noted by researchers. For instance, the removal efficiency of DNA of dead cells is incomplete when small-sized amplicons (<130 bp) and/or high concentration of dead cells are used (Luo et al., 2010; Li and Chen, 2013); and food matrices used may influence the removal efficiency of DNA of dead cells (van Frankenhuyzen et al., 2011). To circumvent the shortcomings of this approach, several strategies can be taken in designing the PMA-qPCR assays: (i) a sensitive, specific and robust qPCR assay is the prerequisite for the development of a sound PMA-qPCR assay (Li and Chen, 2012); (ii) if the target sequence permits, select relatively large amplicon for the PMA-qPCR assay (>130 bp; Li and Chen, 2013); (iii) optimize the PMA treatment conditions such as the concentration of PMA and duration of PMA treatment and light exposure based on different organisms (Gram-positive and Gram-negative; Li and Chen, 2013); and (iv) enhance the PMA’s penetration by adding sodium lauroyl sarcosinate to the PMA solution (Wang et al., 2014; Li et al., 2015). Additionally, selecting alternative chemicals or compounds for PMA or EMA, such as “activity-labile compounds” (Nocker and Camper, 2009) or platinum compounds (Soejima et al., 2016) could be a promising strategy to further improve this viability technology for specific detection of viable pathogens in foods.

## Author Contributions

BL conceived and contributed largely for the review, and DZ and FX contributed in writing, while all other authors contributed in organizing.

## Conflict of Interest Statement

The authors declare that the research was conducted in the absence of any commercial or financial relationships that could be construed as a potential conflict of interest.

## Footnotes

1 https://biotium.com/product/pma-real-time-pcr-bacterial-viability-kit-e-coli-0157h7-z3276/
